# MiR-222-3p Aggravates the Inflammatory Response by Targeting SOCS1 to Activate STAT3 Signaling in Ulcerative Colitis

**DOI:** 10.5152/tjg.2022.21769

**Published:** 2022-11-01

**Authors:** Fei Xia, Wenxia Bo, Jinli Ding, Yanqiu Yu, Jianning Wang

**Affiliations:** Department of Gastroenterology, The Affiliated Jiangning Hospital of Nanjing Medical University, Jiangsu, China

**Keywords:** miR-222-3p, SOCS1, ulcerative colitis, VDR

## Abstract

**Background:**

Ulcerative colitis is characterized by relapsing inflammation in the gastrointestinal tract with limited treatment options. The aim of the present study was to assess the anti-inflammatory effect of Suppressor of cytokine signaling (*SOCS1*) on lipopolysaccharide-stimulated RAW264.7 cells and to investigate its potential mechanisms.

**Methods:**

The in vitro ulcerative colitis model was established by using lipopolysaccharide-stimulated RAW264.7 cells. Western blotting was used to detect the protein expression levels of *SOCS1*, *JAK2*, *STAT3*, and *VDR*. Reverse transcription-quantitative polymerase chain reaction was used to measure the mRNA expression of *SOCS1*, miR-222-3p, and *VDR*. An enzyme-linked immunosorbent assay was performed to measure the levels of inflammatory cytokines. A luciferase assay assessed the binding of SOCS1 to miR-222-3p. A total of 15 patients with ulcerative colitis and 18 healthy controls were recruited. The expression levels of *SOCS1* and miR-222-3p in the colonic mucosa tissues of patients with ulcerative colitis and healthy controls were determined by reverse transcription-quantitative polymerase chain reaction.

**Results:**

*SOCS1 *upregulation inhibited the lipopolysaccharide-induced inflammation in RAW264.7 cells. *SOCS1* was confirmed to be targeted by miR-222-3p. Silencing *SOCS1* significantly abolished the inhibitory effects of miR-222-3p downregulation on inflammation. MiR-222-3p activated STAT3 signaling and reduced *VDR* expression by targeting *SOCS1* in lipopolysaccharide-treated RAW264.7 cells. Additionally, miR-222-3p expression was upregulated in ulcerative colitis patients (*P* = 5.16E−10), while *SOCS1 *(*P* = 2.75E−10) and *VD*R (*P* = 52.5E−9) expression was downregulated in ulcerative colitis patients. Endoscopic scores (UCEIS) revealed significant positive correlation with miR-222-3p and negative correlation with *SOCS1* and *VDR*.

**Conclusion:**

MiR-222-3p targets *SOCS1* to aggravate the inflammatory response by suppressing *VDR *and activating STAT3 signaling in ulcerative colitis.

**Main Points**

- *SOCS1* upregulation inhibits the lipopolysaccharide-induced inflammatory response in RAW264.7 macrophages.
- *SOCS1* is targeted by miR-222-3p.
- MiR-222-3p activates STAT3 signaling and downregulates *VDR* by targeting* SOCS1*.
- MiR-222-3p targets *SOCS1* to aggravate the inflammatory response by activating STAT3 signaling.

## Introduction

Inflammatory bowel disease (IBD) is an intestinal inflammatory disorder encompassing ulcerative colitis (UC) and Crohn’s disease (CD).^1^ The diagnosis rate and hospitalization rate of IBD patients worldwide have been increasing annually over the past decades.^2,3^ Ulcerative colitis is characterized by rectal bleeding, mucosal damage, and ulceration.^4^ Multiple pathogenic factors have been documented, including genetics, immune response, inflammatory factors, bacterial infection, and environmental factors, among which environmental and immunological factors play important roles in UC.^5^ Highly activated monocytes and macrophages in UC patients have been reported.^6^ Activated monocyte/macrophage releases pro-inflammatory cytokines, leading to epithelial barrier disruption and intestinal inflammation.^7,8^ The severity and duration of diffuse mucosal inflammation of the colon are closely related to the risk of colorectal cancer formation.^9^ Therefore, more effective diagnostic and therapeutic targets are needed for early clinical risk assessment and treatment.

Suppressors of cytokine signaling 1 (*SOCS1*) is a key negative regulator of inflammatory processes.^10^ Depletion of *SOCS1* in helper T cells suppresses Th17 differentiation by enhancing the antagonistic effect of IFN-γ.^11^ The proinflammatory genetic background owing to *SOCS1* deficiency causes dysbiosis of the gut microbiota, thereby generating a pro-colitogenic environment.^12^ *SOCS1* relieves dextran sulfate sodium^13^-induced colitis in mice by blocking IFN-γ/STAT1 pathway.^14^ *SOCS1* plays a key role in attenuating murine colitis by inhibiting cytokine signaling and controlling intestinal T cell activation.^15^ These findings demonstrate the important role of *SOCS1* in the pathogenesis of colitis. Additionally, *SOCS1* is a critical negative modulator of JAK/STAT pathway and functions in inhibiting systemic autoimmunity mediated by dendritic cells.^16^ Vitamin D receptor (*VDR*) belongs to the steroid receptor family and its protective effects against colitis have been reported.^17,18^ It has been reported that STAT3 signaling contributes to downregulation of *VDR*.^19^

MicroRNAs (miRNAs) are short non-coding RNAs, which control gene expression by binding to the 3’UTR of mRNAs.^20,21^ Emerging reports have indicated that miRNAs are associated with the procession of UC, such as miR-129-5p, miR-21, and miR-200, suggesting that miRNAs could be therapeutic targets for UC.^22-24^ As previously reported, miR-222-3p expression is upregulated in doxorubicin-resistant colon cancer cells and induces doxorubicin resistance.^25^ Additionally, miR-222-3p depletion alleviates liver inflammatory damage by elevating *SOCS1* expression.^26^ However, the regulatory mechanisms of the miR-222-3p/*SOCS1* in UC are unclear.

In this study, we aimed to investigate the anti-inflammatory effect of* SOCS1* on UC using Raw264.7cells inflammatory model induced by LPS. We also investigated its possible mechanism involving the regulation of JAK/STAT3 signaling and the involvement of miR-222-3p. This study might provide some new potential research targets for molecular mechanisms of UC development.

## Materials and Methods

### Tissue Samples

Colonic mucosa tissues were collected from the sigmoid colon of 15 patients with active UC and 18 healthy controls undergoing screening colonoscopies between June 2015 and July 2016 at The Affiliated Jiangning Hospital of Nanjing Medical University, Nanjing, Jiangsu, China. Informed consent was obtained from all study participants. The inclusion criteria included: active stage of UC, but no prior treatment at the time of serum sampling. Exclusion criteria included: history of autoimmune diseases and previous treatment (immunomodulators, steroids, antibiotics, 5-aminosalicylic acid, blood cell apheresis, biologics, or surgery) at the time of serum sampling. The biopsies were immediately snap-frozen and stored at −80°C after pinching. The study was approved by the Ethics Committee of The Affiliated Jiangning Hospital of Nanjing Medical University, Nanjing, Jiangsu, China.

The endoscopic index was graded following the UCEIS.^27^ The UCEIS includes bleeding, erosions, visible vascular patterns, and ulcers. The UCEIS is stratified into 4 categories: remission (UCEIS 0-1), mild (UCEIS 2-4), moderate (UCEIS 5-6), and severe (UCEIS 7-8).

### Cells

The mouse macrophage RAW264.7 and HEK293T cells (ATCC; Manassas, VA, USA) were cultured in Dulbecco’s modified eagle medium (Gibco, Rockville, USA) containing 10% fetal bovine serum, 100 mg/mL streptomycin, and 100 U/mL penicillin at 37°C with 5% CO_2_. Cells were plated in 96-well plates at 1 × 10^5^/mL at 37°C for 24 hours. Next, cells were cultured with 1 μg/mL LPS (Sigma-Aldrich, St. Louis, MO, USA) at room temperature for 6, 12, and 24 hours to establish an UC model in vitro.

### Transfection

Small interfering RNA targeting *SOCS1* (Si-SOCS1) and Si-NC were constructed by Ribobio (Guangzhou, China). Anti-miR-222-3p and miR-NC were also obtained from Ribobio. Full-length *SOCS1* was amplified and then digested with XbaI to obtain nucleotides of *SOCS1* cDNA sequence and inserted into the pcDNA3.1 vector (Invitrogen, Carlsbad, CA, USA) between EcoRI and EcoRV cleavage sites as the backbone. LPS-cultured cells were transfected with the above vectors using Lipofectamine 2000 (Invitrogen). After 24 hours, the transfection was tested by reverse transcription-quantitative polymerase chain reaction (RT-qPCR).

### Reverse Transcription-Quantitative Polymerase Chain Reaction

Total RNA was extracted from LPS-cultured RAW264.7 cells or specimens utilizing TRIzol (Invitrogen). The extracted RNA was kept at −80°C for later use. Next, cDNA was obtained using a High Capacity cDNA Reverse Transcription kit (Takara, Japan). The primers were synthesized by GenePharma (Shanghai, China), and U6 or GAPDH was as an internal reference. Reverse transcription-quantitative polymerase chain reaction was conducted using a TaqMan MicroRNA Assay kit (Applied Biosystems, Foster City, CA, USA) for miR2223p and a Power SYBR Green qPCR Master Mix (Takara) for *SOCS1*, *VDR*, TNF-α, IL-6, and IL-8 following the manufacturer’s instructions. The expression was evaluated using the 2^−ΔΔCt^. The mouse primers used are shown in Table 1.

### Western Blotting

Total proteins were collected from specimens in ice-cold RIPA lysis buffer (Beyotime, China). Next, a bicinchoninic acid kit (Soliba, China) was utilized to determine the protein concentration. Total proteins (40 μg) were separated by 12% SDS-PAGE and blotted onto nitrocellulose membranes. The membranes were blocked in Tris-buffered saline and 5% nonfat milk for 1 hour, and then incubated overnight at 4°C with antibodies including *SOCS1* (ab280886, Abcam, Cambridge, UK), P-JAK2 (ab32101), JAK2 (ab32101), p-STAT3 (ab76315), STAT3 (ab68153), *VDR *(ab109234), and GAPDH (ab9485), and further probed with peroxidase-labeled goat anti-rabbit (Santa Cruz Biotechnology, CA, USA). The membranes were washed before detection and observed utilizing an enhanced chemiluminescence method (Amersham Biosciences, Arlington Heights, IL, USA). Signal quantification was performed with ImageJ software.

### Enzyme-Linked Immunosorbent Assay

The inflammatory cytokine (TNF-α, IL-6, and IL-8) levels in the supernatant were determined using commercial mouse-specific enzyme-linked immunosorbent assay (ELISA) kits (Abcam). Culture supernatant was obtained from the transfected RAW264.7 following LPS treatment at the indicated time. The absorbance was measured using a Universal Microplate Spectrophotometer (BioTek Instruments, Winooski, VT, USA). The obtained results were expressed as picograms per milliliters of protein.

### Luciferase Reporter Assay

The binding site of miR-222-3p in *SOCS1* 3’UTR was predicted at the TargetScan website. The *SOCS1* 3’UTR fragment at the miR-222-3p binding site was synthesized to generate *SOCS1*-Wt plasmids. *SOCS1*-Mut plasmids were constructed by mutating the miR-222-3p binding site in *SOCS1*-Wt. 293T cells were seeded in 96-well plates until reached 70%, and Lipofectamine 2000 was used for transfection. The *SOCS1*-Wt/Mut plasmids were co-transfected into 293T cells with miR-NC (75 nM) or Anti-miR-222-3p (75 nM). After 48 hours, cells were lysed for analyses using Luciferase Reporter Assay System (Promega, Madison, WI, USA).

### Statistical Analysis

Each assay was conducted at least 3 times to prevent errors. GraphPad Prism 6 software (GraphPad Software Inc., San Diego, CA, USA) was used for statistical analyses. Values are expressed as mean ± standard deviation. The Student’s *t*-test was used for data analysis of pairwise comparison. Comparison between 3 or more groups was analyzed by one-way ANOVA. Correlations between the UCEIS and gene expression were analyzed using nonparametric Spearman’s correlation. *P *< .05 was considered statistically significant.

## Results

### Lipopolysaccharide Induces Inflammatory Response and Downregulates ***SOCS1***

**Figure 1.**
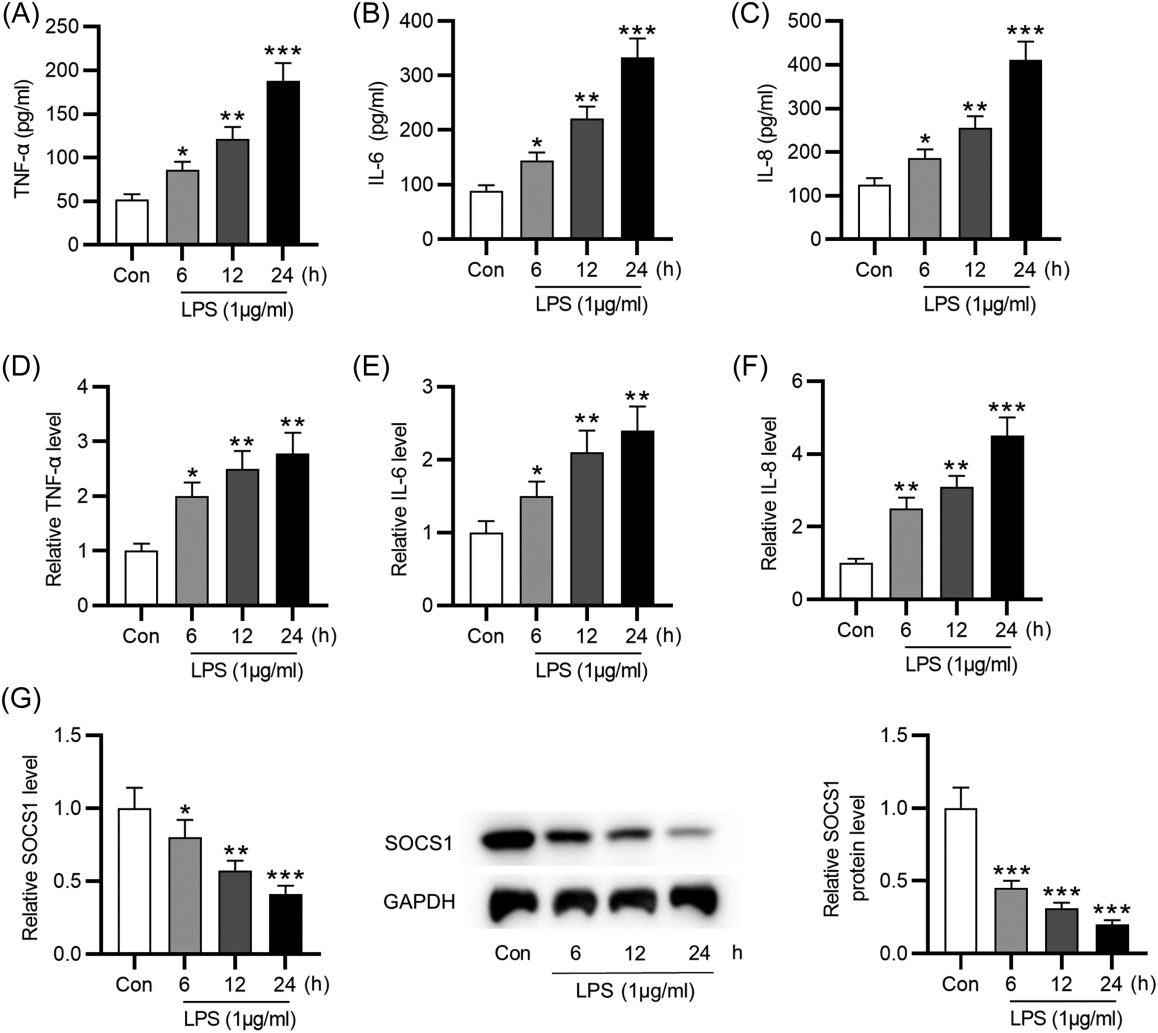
LPS induces inflammatory response and downregulates *SOCS1*. The concentrations of TNF-α (A), IL-6 (B), and IL-8 (C) in LPS-treated RAW264.7 cells were detected by ELISA. The mRNA expression of TNF-α (D), IL-6 (E), and IL-8 (F) was detected by RT-qPCR. (G) *SOCS1* mRNA and protein expression was measured by RT-qPCR and western blotting. ^*^ *P *< .05, ^**^ *P *< .01, ^***^ *P *< .001. LPS, lipopolysaccharide; ELISA, enzyme-linked immunosorbent assay.

We established inflammatory response models in RAW264.7 cells using LPS. The ELISA results showed that LPS notably elevated the concentrations of TNF-α (at 24 hours: 188 ± 20.01; *P* = 7.55E−6), IL-6 (at 24 hours: 333 ± 35.37; *P* = 4.98E−6), and IL-8 (at 24 hours: 411 ± 42.04; *P* = 6.8E−6) in RAW264.7 cells in a time-dependent manner (Figure 1A-C). Their mRNA levels were detected by RT-qPCR. The data indicated that the mRNA levels of TNF-α (at 24 hours: 2.78 ± 0.33; *P* = 2.05E−4), IL-6 (at 24 hours: 2.4 ± 0.33; *P* = .001), and IL-8 (at 24 hours: 4.5 ± 0.5; *P* = 5.74E−6) in RAW264.7 cells were upregulated after LPS treatment (Figure 1D-F). Subsequently, the *SOCS1* level in LPS-cultured RAW264.7 cells was evaluated by RT-qPCR and western blotting. The findings revealed that *SOCS1* mRNA (at 24 hours: 0.41 ± 0.06; *P* = .001) and protein (at 24 hours: 0.2 ± 0.03; *P* = 3.44E−6) expression was decreased after LPS treatment (Figure 1G).

### Effects of ***SOCS1*** on Inflammation of Macrophages

**Figure 2.**
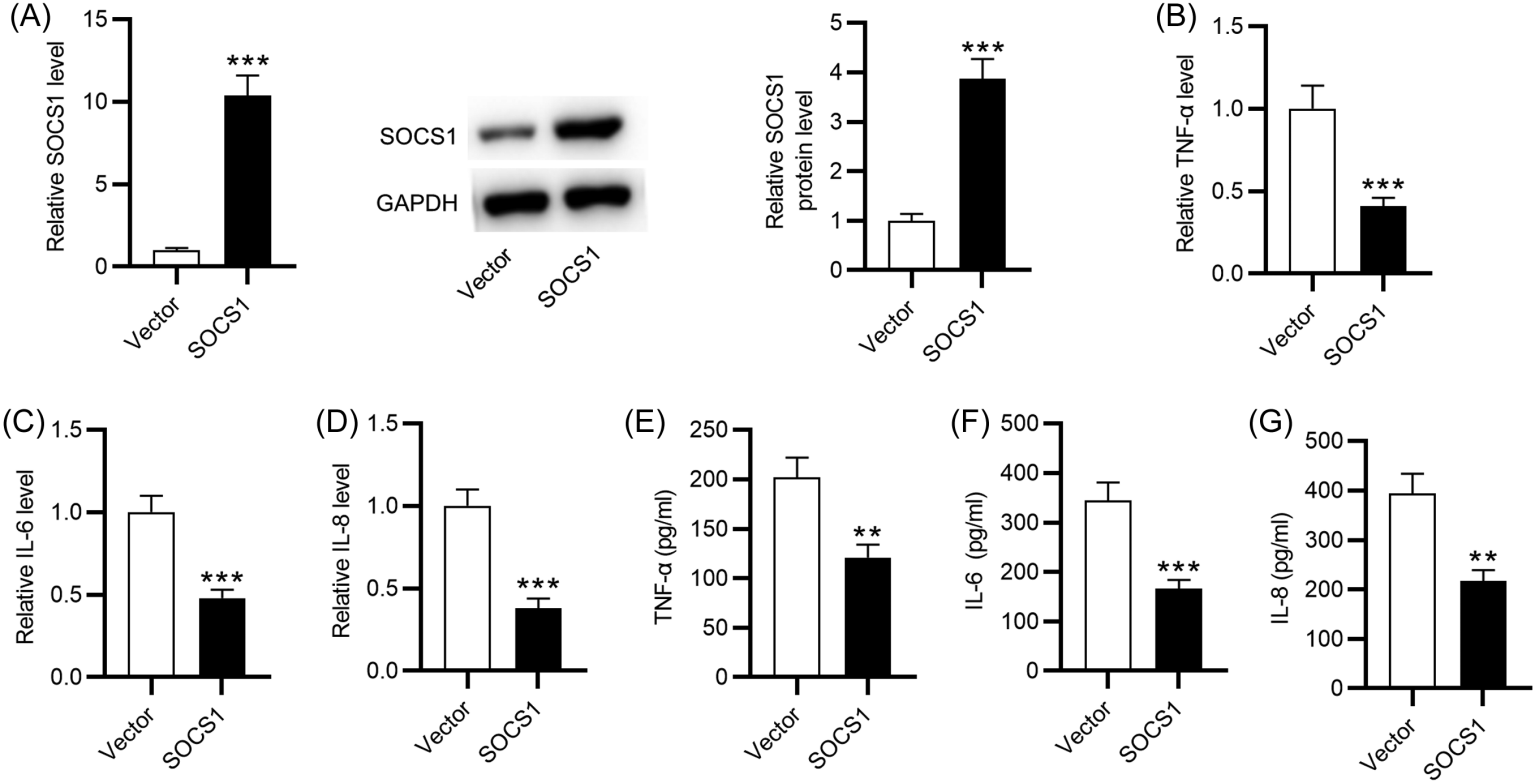
Effects of* SOCS1* on inflammation of macrophages. (A) *SOCS1* mRNA and protein expression in LPS-treated RAW264.7 cells transfected with *SOCS1* or vector was measured by RT-qPCR and western blotting. The mRNA expression of TNF-α (B), IL-6 (C), and IL-8 (D) after transfection with *SOCS1* or vector was detected by RT-qPCR. The concentrations of TNF-α (E), IL-6 (F), and IL-8 (G) in LPS-treated RAW264.7 cells transfected with *SOCS1 *or vector were detected by ELISA.^ **^ *P *< .01, ^***^ *P *< .001. LPS, lipopolysaccharide, RT-qPCR, reverse transcription-quantitative polymerase chain reaction.

We further tested the role of *SOCS1* in UC. In Figure 2A, the* SOCS1* mRNA (10.4 ± 1.18; *P* = 1.65E−4) and protein (3.87 ± 0.4; *P* = 3.0E−4) expression in RAW264.7 cells was upregulated after transfection with *SOCS1*, indicating the successful transfection. Then, the levels of cytokines in LPS-treated RAW264.7 cells were detected after* SOCS1* was overexpressed. *SOCS1* upregulation significantly downregulated the mRNA levels of TNF-α (0.41 ± 0.05; *P* = .002), IL-6 (0.48 ± 0.09; *P* = .004), and IL-8 (0.38 ± 0.06; *P* = .001) (Figure 2B-D). Similarly, the supernatant levels of TNF-α (121 ± 12.6; *P* = .004), IL-6 (166 ± 17.84; *P* = .002), and IL-8 (217.09 ± 22; *P* = .003) were reduced after overexpressing *SOCS1 *in RAW264.7 cells (Figure 2E-G). Overall, *SOCS1* upregulation inhibited the LPS-induced inflammatory response.

### ***SOCS1*** is Targeted by miR-222-3p

**Figure 3.**
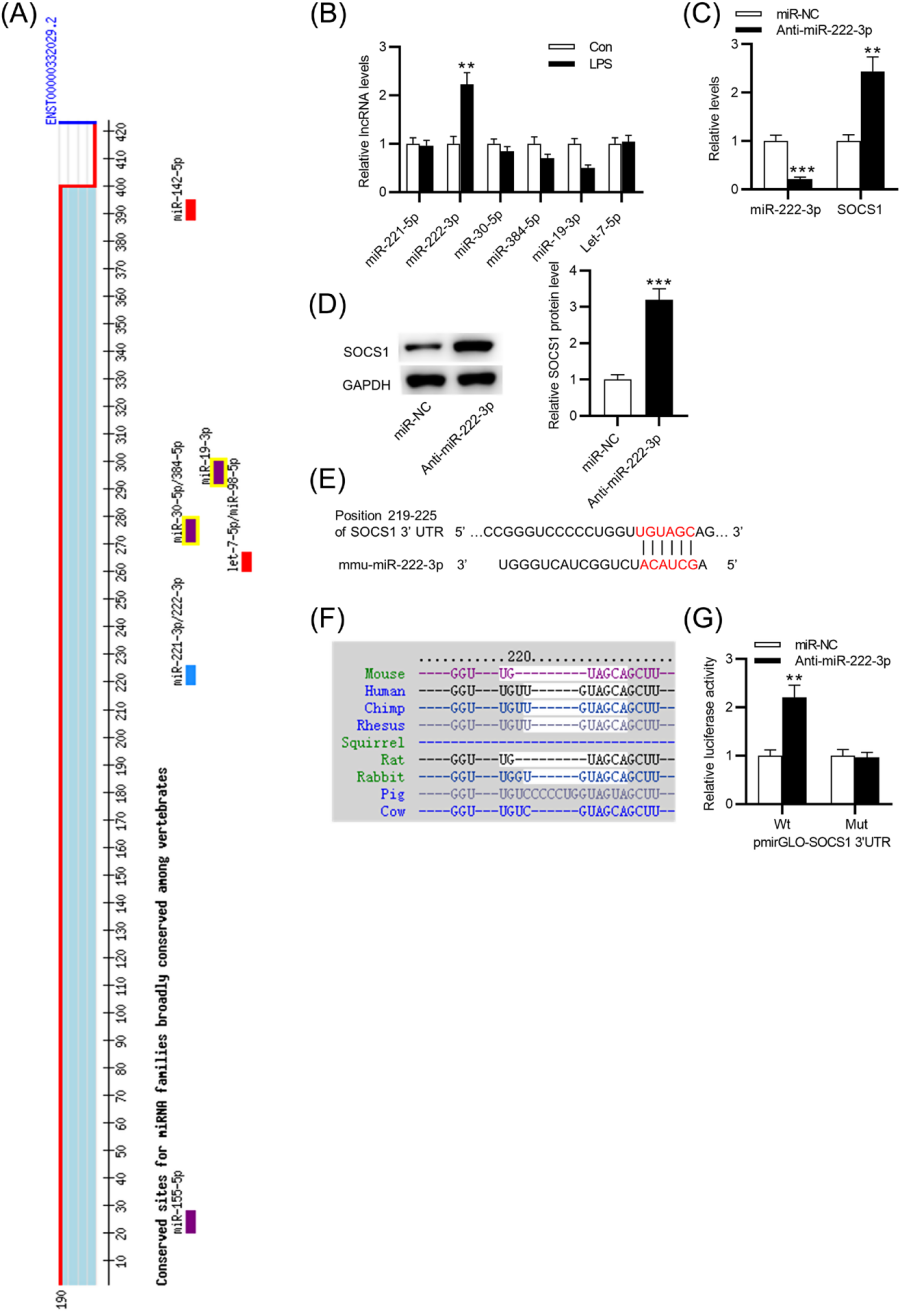
*SOCS1* is targeted by miR-222-3p. (A) TargetScan prediction results show 9 miRNAs that have conserved sites for *SOCS1*. (B) The miRNA expression in LPS-treated RAW264.7 cells was measured by RT-qPCR. (C) MiR-222-3p expression and *SOCS1* expression in RAW264.7 cells transfected with anti-miR-222-3p or control. (D) *SOCS1* protein expression in RAW264.7 cells transfected with anti-miR-222-3p or control. (E-F) Position 219-225 of *SOCS1* 3’UTR in miR-222-3p is shown highly conserved in multiple species. (G) The luciferase activity of* SOCS1* 3’UTR in 293T cells transfected with anti-miR-222-3p or control was measured by luciferase reporter assay. ^**^ *P *< .01, ^***^ *P *< .001. LPS, lipopolysaccharide, RT-qPCR, reverse transcription-quantitative polymerase chain reaction.

TargetScan was utilized to identify the miRNAs binding to *SOCS1*. The prediction results show 9 miRNAs that have conserved sites for* SOCS1* (Figure 3A), among which miR-155-5p, miR-142-5p, and miR-98-5p were reported in IBD. Therefore, we measured the levels of the other 6 miRNAs in LPS-treated RAW264.7 and uncovered that miR-222-3p was significantly upregulated (2.23 ± 0.24; *P* = .002) (Figure 3B), showing the potential role of miR-222-3p in UC. Next, RAW264.7 cells were transfected with Anti-miR-222-3p. As revealed in Figure 3C, miR-222-3p was downregulated (0.21 ± 0.04; *P* = 4.34E−4) and *SOCS1* was upregulated (2.44 ± 0.33; *P* = .002) after transfection with Anti-miR-222-3p. Additionally, *SOCS1* protein levels were increased after miR-222-3p was downregulated (3.2 ± 0.3; *P* = 3.01E−4) (Figure 3D), demonstrating that miR-222-3p negatively modulated *SOCS1*. Position 219-225 of *SOCS1* 3’UTR in miR-222-3p is presented in Figure 3E, and this binding site is highly conserved in multiple species (Figure 3F). Furthermore, this prediction was demonstrated by a luciferase reporter assay. As suggested in Figure 3G, depletion of miR-222-3p increased the luciferase activity of the *SOCS1* 3’UTR-Wt group in 293T cells (2.21 ± 0.25; *P* = .002). Therefore, *SOCS1 *was targeted by miR-222-3p.

### ***SOCS1*** Knockdown Eliminates the Effects of miR-222-3p Inhibition in LPS-Treated RAW264.7 Cells

**Figure 4.**
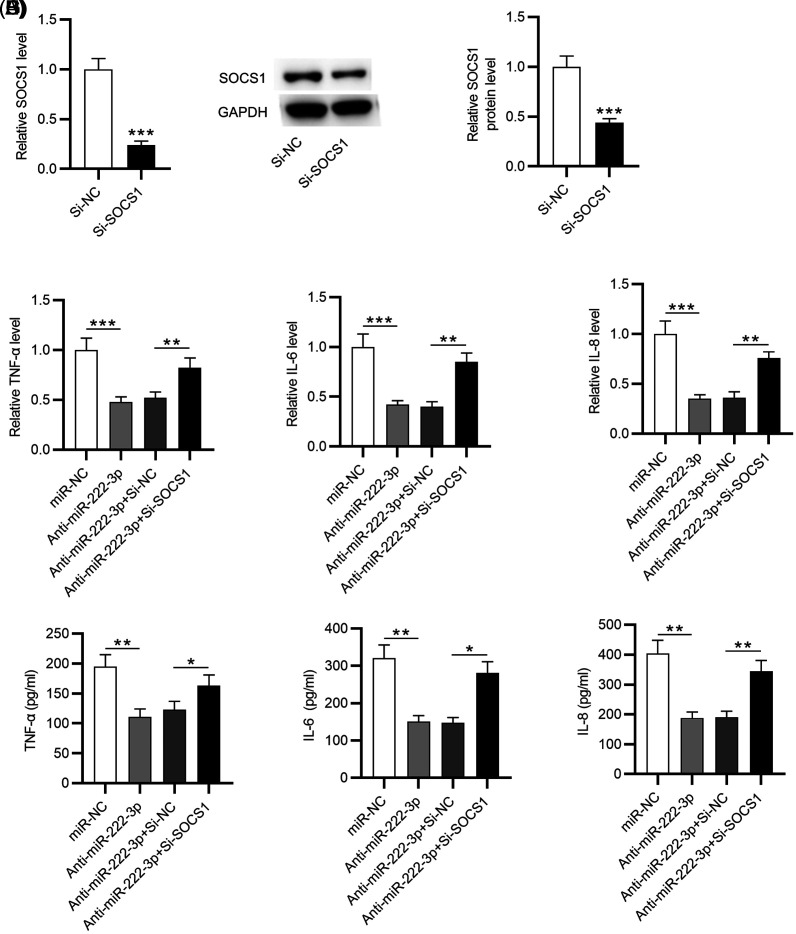
*SOCS1* knockdown eliminates the effects of miR-222-3p inhibition. (A-B) *SOCS1* mRNA and protein expression in LPS-treated RAW264.7 cells transfected with Si-SOCS1 or NC was measured by RT-qPCR and western blotting. The mRNA expression of TNF-α (C), IL-6 (D), and IL-8 (E) in LPS-treated RAW264.7 cells was detected by RT-qPCR. The concentrations of TNF-α (F), IL-6 (G), and IL-8 (H) in LPS-treated RAW264.7 cells were detected by ELISA.^ *^ *P *< .05, ^**^ *P *< .01, ^***^ *P *< .001. LPS, lipopolysaccharide, RT-qPCR, reverse transcription-quantitative polymerase chain reaction; ELISA, enzyme-linked immunosorbent assay.

Reverse transcription-quantitative polymerase chain reaction and western blotting revealed that *SOCS1* mRNA (0.24 ± 0.04; *P* = 3.54E−4) and protein (0.44 ± 0.04; *P* = .001) expression was decreased after transfection with Si-SOCS1 (Figure 4A and B). RAW264.7 cells were then transfected with the indicated plasmids and exposed to 1 μg/mL LPS for 24 hours. The mRNA expression and concentrations of cytokines in the culture supernatant were measured. In Figure 4C-E, miR-222-3p depletion reduced the mRNA expression of TNF-α (0.48 ± 0.05; *P* = 3.61E−4), IL-6 (0.42 ± 0.05; *P* = 1.24E−4), and IL-8 (0.35 ± 0.04; *P* = 4.32E−5), as well as reduced the concentrations of TNF-α (123 ± 14; *P* = .003), IL-6 (151 ± 16; *P* = 1.75E−4), and IL-8 (188 ± 20.03; *P* = 1.41E−4) in LPS-cultured RAW264.7 cells (Figure 4F-H). Moreover,* SOCS1* depletion significantly abolished the inhibitory effects of reduced miR-222-3p on inflammation induced by LPS.

### MiR-222-3p Activates STAT3 Signaling and Reduces ***VDR*** Expression by Targeting ***SOCS1***

**Figure 5.**
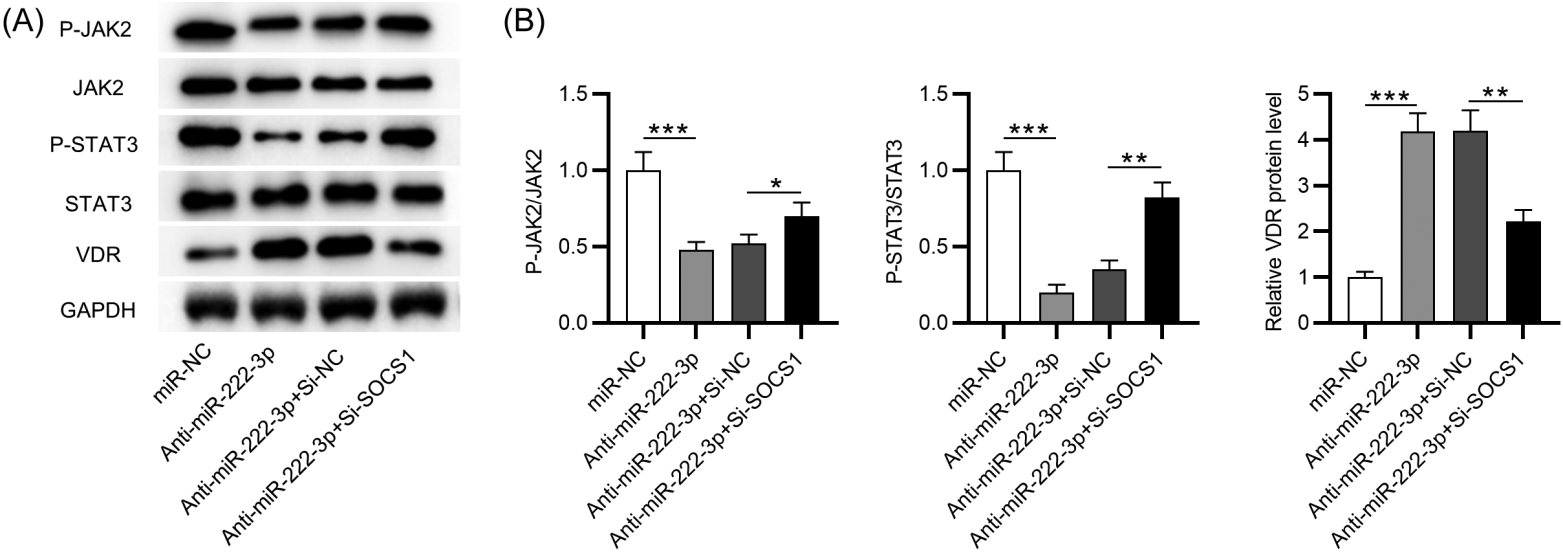
miR-222-3p activates STAT3 signaling and reduces *VDR* expression by targeting *SOCS1*. (A-B) The protein expression of P-JAK2/JAK2, P-STAT3/STAT3, and *VDR *in LPS-treated RAW264.7 cells was detected by western blotting. ^*^ *P *< .05, ^**^ *P *< .01, ^***^ *P *< .001.

Subsequently, the effect of the miR-222-3p/*SOCS1* axis in STAT3 signaling under inflammatory conditions was analyzed using western blotting. Inhibition of miR-222-3p reduced the P-JAK2 (0.48 ± 0.05; *P* = 2.6E−4) and P-STAT3 (0.2 ± 0.05; *P* = 2.32E−5) protein expression while increased the *VDR* (4.18 ± 0.4; *P* = 7.58E−6) protein expression, and these effects were reversed after *SOCS1 *depletion (Figure 5A and B), indicating that silencing miR-222-3p suppresses STAT3 signaling and upregulates *VDR* expression by upregulating *SOCS1*.

### Correlations Between Molecule Expression and Endoscopic Scores (UCEIS) in UC Patients

**Figure 6.**
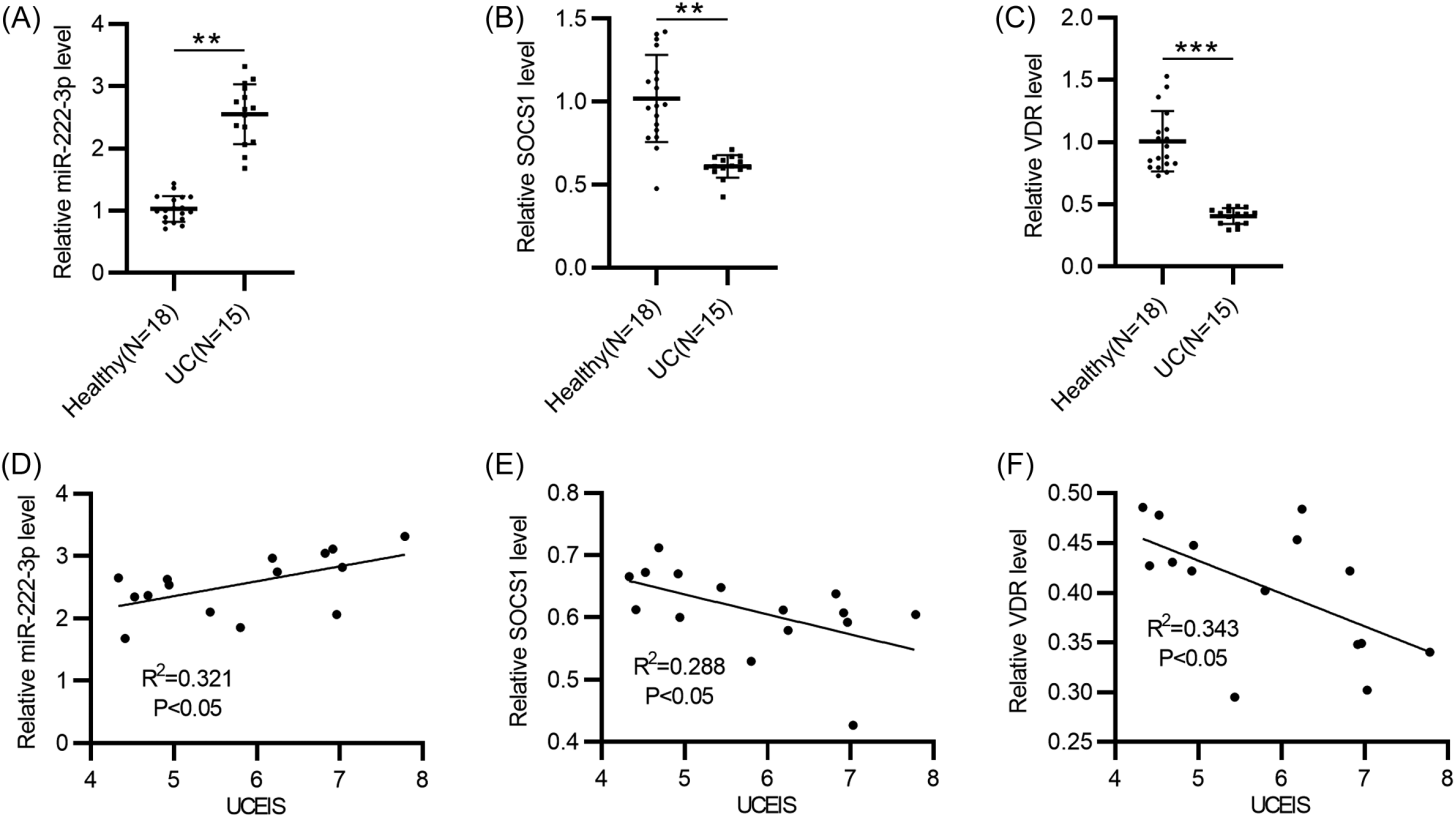
Correlations between molecule expression and endoscopic scores (UCEIS) in UC patients. Comparison of miR-222-3p (A), *SOCS1* (B), and *VDR* (C) expression between healthy controls and UC patients. The levels of (D) miR-222-3p, (E) *SOCS1*, and (F) *VDR* were correlated with the endoscopic score of UC (UCEIS). Correlations were assessed by Spearman’s correlation coefficient. ^**^ *P *< .01, ^***^ *P *< .001. UC, ulcerative colitis.

The colonic mucosa tissue levels of miR-222-3p, *SOCS1*, or *VDR* between UC patients (n = 15) and healthy controls (n = 18) were compared. As indicated in Figure 6A-C, miR-222-3p (2.55 ± 0.48; *P* = 5.16E−10) expression was higher in UC patients than in healthy controls, while *SOCS1* (0.61 ± 0.07; *P* = 2.75E−6) and* VDR *(0.41 ± 0.06; *P* = 2.5E−9) expression was lower in UC patients. Additionally, the UCEIS revealed a significant positive correlation with miR-222-3p (R^2^ = 0.321, *P* = .03), and negative correlation with *SOCS1* (R^2^ = 0.288, *P* = .006) and *VDR* (R^2^ = 0.343, *P* = .02).

## Discussion

*SOCS1* was found downregulated in colonic mucosa tissue samples of UC and TNF-α-stimulated human intestinal epithelial cells.^28^ Here, we demonstrated that *SOCS1* expression showed a decreased trend in murine macrophage cells after LPS treatment. However, a study indicated that the expression of *SOCS1 *was seen to increase in the active UC population.^29^ We hypothesize that different population of samples may turn out differently. Although UC can be triggered by bacteria, viruses, and other environmental factors, the inflammatory process of the intestinal mucosa is ultimately induced by soluble inflammatory mediators.^30^ The inflammatory mediators such as IL-1beta and TNF-α play leading roles in the development of UC.^31^ Therefore, the effective reduction of inflammatory mediators in the serum and colon tissues is a reasonable modality for UC treatment.^32,33^ Additionally, the anti-inflammatory effects of *SOCS1* in immune disease have been uncovered. *SOCS1* inhibits its downstream toll-like receptor-mediated inflammatory pathways in RAW264.7 macrophages.^34^ *SOCS1* production is induced by resveratrol in LPS-cultured RAW264.7 macrophages to inhibit the release of TNF-α and IL-6.^35^ Moreover, silencing *SOCS1* in the cell population that contributes to mucosal damage in IBD increases IL-6 and IL-8 production.^36^ *SOCS1* was also shown involved in the inflammatory mechanisms in the colonic mucosa of UC.^37^ In this study, our experiments further demonstrated that* SOCS1* inhibited the release of inflammatory cytokines in LPS-stimulated RAW264.7 macrophages. Moreover, *SOCS1 *expression was downregulated in UC patients.

Emerging evidence has demonstrated that miRNAs are implicated in the pathogenesis of UC. Loss of miR-24-3p promotes epithelial cell apoptosis and impairs the recovery from intestinal inflammation.^38^ MiR-200b-3p alleviates TNF-α-induced apoptosis and inflammation of intestinal epithelial cells and ulcerative colitis progression in rats.^39^ Here, we identified that miR-222-3p has a binding site for *SOCS1 *by bioinformatics analysis and confirmed their targeted relationship. As reported, miR-222-3p is overexpressed in inflammatory breast cancer as well as in children with mycoplasma pneumonia, acting as a promising biomarker for the diagnosis of diseases.^40,41^ The role of miR-222-3p in UC is unknown. Here, we showed high expression of miR-222-3p in LPS-treated RAW264.7 macrophages and in UC patients. Moreover, miR-222-3p silencing showed inhibitory effects on LPS-stimulated inflammation, while these effects were reversed by *SOCS1* depletion, suggesting the effects of the miR-222-3p/*SOCS1* axis in UC.

JAK/STAT components could be activated by a series of cytokines related to receptor families, including the gp130 receptor family, such as IL-6.^42^ STAT3 activation leads to pro-inflammatory activities including Th17 differentiation in lymphocytes,^43,44^ suggesting the strong plasticity of JAK/STAT pathway in the regulation of inflammatory responses. Moreover, JAK/STAT signaling activation shows a strong inflammatory involvement in the pathogenesis of UC.^45^ It has been indicated that assessment of specific genotypes of JAK/STAT inhibition could be an effective treatment idea for UC.^46^ The JAK/STAT inhibitor tofacitinib has been recently approved for the treatment of active UC.^45^ Therefore, investigation of JAK/STAT in UC may contribute to a better understanding of UC pathogenesis. *SOCS1* is a negative modulator of JAK/STAT signaling through degradation of JAK2 or suppression of JAK1 and JAK2.^47,48^ We examined the effects of the miR-222-3p/*SOCS1* axis on STAT3 signaling under inflammatory conditions and found that silencing miR-222-3p suppressed STAT3 signaling by upregulating* SOCS1*. Additionally, dysfunction of *VDR* contributes to the etiology of IBD by regulating autophagy, immune response, and mucosal permeability.^49^ *VDR* is shown to have a protective effect on the onset or progression of IBD.^50^ It was reported that STAT signaling contributes to the downregulation of *VDR*.^19^ In this investigation, miR-222-3p depletion downregulated *VDR* in LPS-stimulated RAW264.7 cells by upregulating *SOCS1*.

In conclusion, we revealed that miR-222-3p expression was high in UC patients, while *SOCS1* and *VDR* expression was low in UC patients. In addition, miR-222-3p targeted *SOCS1* to aggravate the inflammatory response by suppressing *VDR* and activating STAT3 signaling in UC. It may provide a new regulatory mechanism for *SOCS1* in the investigation of UC progression. However, there were some limitations in the present study. These included the small number of samples and specimens from the same area. The overall results were statistically significant, but whether miR-222-3p/*SOCS1* can be used as a reliable diagnostic indicator of UC and can be used clinically requires further research and verification. These findings should be confirmed and evaluated in prospective, large-sample, multicenter, randomized clinical trials.

## Figures and Tables

**Table 1.** Primer sequences used for RT-qPCR

Target	Primer sequences (5′–3′)
SOCS1	Forward, CTTCTGTAGGATGGTAGCACAC
SOCS1	Reverse, AGGAAGAGGAGGAAGGTTCT
miR-222-3p	Forward, GGGGAGCTACATCTGGCT
miR-222-3p	Reverse, TGCGT GTCGTGGAGTC
IL-6	Forward, TCCAGTTGCCTTCTTGGGACTG
IL-6	Reverse, AGCCTCCGACTTGTGAAGTGGT
IL-8	Forward, ATGACTTCCAAGCTGGCCGT
IL-8	Reverse, TTACATAATTTCTGTGTTGGC
TNF-α	Forward, GACCCTCACACTCAGATCATCT
TNF-α	Reverse, CCTCCACTTGGTGGTTTGCT
VDR	Forward, GAATGTGCCTCGGATCTGTGG
VDR	Reverse, ATGCGGCAATCTCCATTGAAG
GAPDH	Forward, AGGTCGGTGTGAACGGATTTG
GAPDH	Reverse, GGGGTCGTTGATGGCAACA
U6	Forward, CTCGCTTCGGCAGCACA
U6	Reverse, AACGCTTCACGAATTTGCGT

RT-qPCR, reverse transcription-quantitative polymerase chain reaction.
